# Detection of diabetic patients in people with normal fasting glucose using machine learning

**DOI:** 10.1186/s12916-023-03045-9

**Published:** 2023-09-07

**Authors:** Kun Lv, Chunmei Cui, Rui Fan, Xiaojuan Zha, Pengyu Wang, Jun Zhang, Lina Zhang, Jing Ke, Dong Zhao, Qinghua Cui, Liming Yang

**Affiliations:** 1 Key Laboratory of Non-Coding RNA Transformation Research of Anhui Higher Education Institutes, Wuhu, China; 2 Central Laboratory, First Affiliated Hospital of Wannan Medical College, Wuhu, People’s Republic of China; 3 Department of Biomedical Informatics, State Key Laboratory of Vascular Homeostasis and Remodeling, School of Basic Medical Sciences, Peking University, Beijing, People’s Republic of China; 4 Laboratory Medicine, First Affiliated Hospital of Wannan Medical College, Wuhu, People’s Republic of China; 5 Department of Pathophysiology, Harbin Medical University, Harbin, People’s Republic of China; 6 Medical College of Shihezi University, Shihezi, People’s Republic of China; 7 Department of Laboratory Diagnosis, Daqing Oil Field General Hospital, Daqing, People’s Republic of China; 8 Beijing Key Laboratory of Diabetes Research and Care, Center for Endocrine Metabolism and Immune Diseases, Beijing Luhe Hospital, Capital Medical University, Beijing, People’s Republic of China; 9 National Key Laboratory of Frigid Zone Cardiovascular Diseases (NKLFZCD), Harbin Medical University, Harbin, People’s Republic of China; 10 NHC Key Laboratory of Cell Transplantation, The First Affiliated Hospital of Harbin Medical University, Harbin, People’s Republic of China

**Keywords:** Diabetes risk prediction, Normal fasting glucose, Machine learning, Missed diagnosis

## Abstract

**Background:**

Diabetes mellitus (DM) is a chronic metabolic disease that could produce severe complications threatening life. Its early detection is thus quite important for the timely prevention and treatment. Normally, fasting blood glucose (FBG) by physical examination is used for large-scale screening of DM; however, some people with normal fasting glucose (NFG) actually have suffered from diabetes but are missed by the examination. This study aimed to investigate whether common physical examination indexes for diabetes can be used to identify the diabetes individuals from the populations with NFG.

**Methods:**

The physical examination data from over 60,000 individuals with NFG in three Chinese cohorts were used. The diabetes patients were defined by HbA1c ≥ 48 mmol/mol (6.5%). We constructed the models using multiple machine learning methods, including logistic regression, random forest, deep neural network, and support vector machine, and selected the optimal one on the validation set. A framework using permutation feature importance algorithm was devised to discover the personalized risk factors.

**Results:**

The prediction model constructed by logistic regression achieved the best performance with an AUC, sensitivity, and specificity of 0.899, 85.0%, and 81.1% on the validation set and 0.872, 77.9%, and 81.0% on the test set, respectively. Following feature selection, the final classifier only requiring 13 features, named as DRING (diabetes risk of individuals with normal fasting glucose), exhibited reliable performance on two newly recruited independent datasets, with the AUC of 0.964 and 0.899, the balanced accuracy of 84.2% and 81.1%, the sensitivity of 100% and 76.2%, and the specificity of 68.3% and 86.0%, respectively. The feature importance ranking analysis revealed that BMI, age, sex, absolute lymphocyte count, and mean corpuscular volume are important factors for the risk stratification of diabetes. With a case, the framework for identifying personalized risk factors revealed FBG, age, and BMI as significant hazard factors that contribute to an increased incidence of diabetes. DRING webserver is available for ease of application (http://www.cuilab.cn/dring).

**Conclusions:**

DRING was demonstrated to perform well on identifying the diabetes individuals among populations with NFG, which could aid in early diagnosis and interventions for those individuals who are most likely missed.

**Supplementary Information:**

The online version contains supplementary material available at 10.1186/s12916-023-03045-9.

## Background

Diabetes mellitus (DM) is one of major public health threat in twenty-first century, characterized by signs of hyper-glycaemia and long-time accompanied with that would cause severe damage to multiple organs including but not limited to the heart, kidney, foot, and retinal peripheral nerve [1]. As estimated by the International Diabetes Federation (IDF), there exists around 537 million people with diabetes in 2021, and the number will rise to 643 million by 2030 [2]. The subjects with diabetes are usually asymptomatic at preliminary stage, which is why the diagnosis for diabetes is often delayed, thus causing serious complications and even fatal reactions. A recent study has revealed that almost 50% patients with newly diagnosed diabetes have the clinical manifestations of micro- and macrovascular disease [3]. This suggests that those individuals with diabetes might remain undiagnosed or untreated for several years, which certainly exacerbates the economic burden on both patients and the healthcare system [4, 5]. It is projected that the cost of per diabetes individual per year would increase from $231 to $414 during 2020–2030, companied with that the total costs of diabetes would elevate from $250.2 billion to $460.4 billion in China [6]. Moreover, the healthcare costs for diabetes patients with complications are more than two times higher compared to those without complications [7, 8]. Therefore, early detection of diabetes is critical for preventing the sustained progression of diabetes, and the onset of associated complications and correspondingly cost-effective strategies for diagnosis of diabetes are warranted.

Considering the cost- and time-consuming of detecting multiple characteristics for diabetes diagnosis in large scale population, especially for some developing countries with rapidly rising prevalence of diabetes, it is an effectively alternative strategy to develop a predictive model for screening the individuals at high risk of diabetes for further examination. Improvement in the data amount advances the application of machine learning on diabetes. At present, increasing computational methods have been proposed for the prediction of diabetes risk using conventional statistical analysis or machine learning methods and achieved promising performance on the risk stratification of diabetes [9–14]. A diabetes risk score (DRS) based on the mainland Chinese population was constructed by multivariable stepwise logistic regression and achieved the AUC of 0.828 and 0.909 for detecting abnormal glucose tolerance and diabetes [11]. Mani et al. applied six machine learning methods combined with electronic medical record to predict the risk of development of diabetes 6 months and 1 year later, in which AUCs were both greater than 0.8 [15]. Among the various machine learning methods, support vector machine, random forest, and ensemble classifier have been shown to produce better classification outcomes [16]. And risk factors relevant to diabetes including age, family history of diabetes, blood pressure, body mass index (BMI), lipoprotein, cholesterol, fasting blood glucose (FBG), the questionnaire on medical history and exercise, and other physical examination indexes are frequently utilized [17]. For risk stratification, the most fundamental issue is to assign a correct class for each sample, here referred to diabetes or health, before constructing the prediction model. The utilization of diabetes definition criteria often varies among different studies, with common diagnostic indicators including FBG, oral glucose tolerance test, and glycated hemoglobin (HbA1c) [18, 19]. However, it has been reported that FBG used for diabetes diagnosis could grossly underestimates the prevalence of diabetes [20]. This implies that some subjects with diabetes might show normal fasting glucose (NFG) level (e.g., the threshold of 6.1 mmol/L according to World Health Organization (WHO) [21]) and thus be at high risk of being missed. Currently, most prediction models are designed to distinguish between individuals with impaired fasting glucose or diabetes and normal individuals; it is hard for them to accurately identify the diabetes individuals with NFG from a large population especially for models using FBG as the definition criterion for diabetes. Therefore, it is quite important and necessary to precisely and efficiently detect these undiagnosed diabetic patients in the people with NFG for timely and early diabetes prevention and treatment.

In the present study, we proposed a machine learning model, DRING (diabetes risk of individuals with normal fasting glucose), for identifying the missed diabetes patients from the individuals with NFG based on physical examination data from more than 60,000 individuals. With feature selection, DRING performed well on validation set and multiple test sets including newly recruited test sets. Moreover, DRING can also uncover the risk factors of diabetes using feature importance analysis. Finally, we devised a framework for screening the personalized risk factors of diabetes and demonstrated its practicability and reliability on a case study.

## Methods

### Data collection and processing

The physical examination data were derived from three hospitals, First Affiliated Hospital of Wannan Medical College, Beijing Luhe Hospital of Capital Medical University, and Daqing Oil field General Hospital. The three datasets were named as D1, D2, and D3, respectively. The first step was data cleaning, in which samples with missing values and abnormal values were excluded. According to the criteria for diagnosing prediabetes and diabetes from WHO, we screened the samples with normal fasting glucose (≤ 6.1 mmol/L) and classified these samples into two groups by HbA1c level with threshold of 6.5%, diabetes patients (HbA1c ≥ 6.5%) and normal/healthy samples. After preprocessing, 61,059, 369, and 3247 samples were retained in D1, D2, and D3, which separately contained 603, 3, and 21 subjects with diabetes, that is, the positive samples. Then, we split D1 into training set, validation set, and test set by 6:1:3 using randomly stratified sampling. D2 and D3 were used as newly recruited independent test sets.

All datasets contained 27 physical examination characteristics, including sex, age, height, body mass index (BMI), fasting blood glucose (FBG), white blood cell count (WBC), neutrophil (NEU), absolute neutrophil count (ANC), lymphocyte (LYM), absolute lymphocyte count (ALC), monocyte (MONO), absolute monocyte count (AMC), eosinophil (EOS), absolute eosinophil count (AEC), basophil (BASO), absolute basophil count (ABC), hemoglobin (HGB), hematocrit (HCT), mean corpuscular volume (MCV), mean corpuscular hemoglobin (MCH), red cell distribution width (RDW), platelets (PLT), mean platelet volume (MPV), platelet distribution width (PDW), thrombocytopenia (PCT), red blood cell count (RBC), and mean corpuscular hemoglobin concentration (MCHC).

Given the severe class-imbalance of all datasets, the SMOTE (synthetic minority over-sampling technique) method was employed on training set for oversampling the positive samples. SMOTE could generate new samples for the minority class by interpolation based on k-nearest neighbors [22], which could make positive samples as large as negative samples on training set. The process was implemented by “imblearn” package in Python. Finally, we conducted *Z*-score normalization on all datasets, in which the mean and standard deviation values were calculated by the data of training set.

### The framework of DRING

**Fig. 1:**
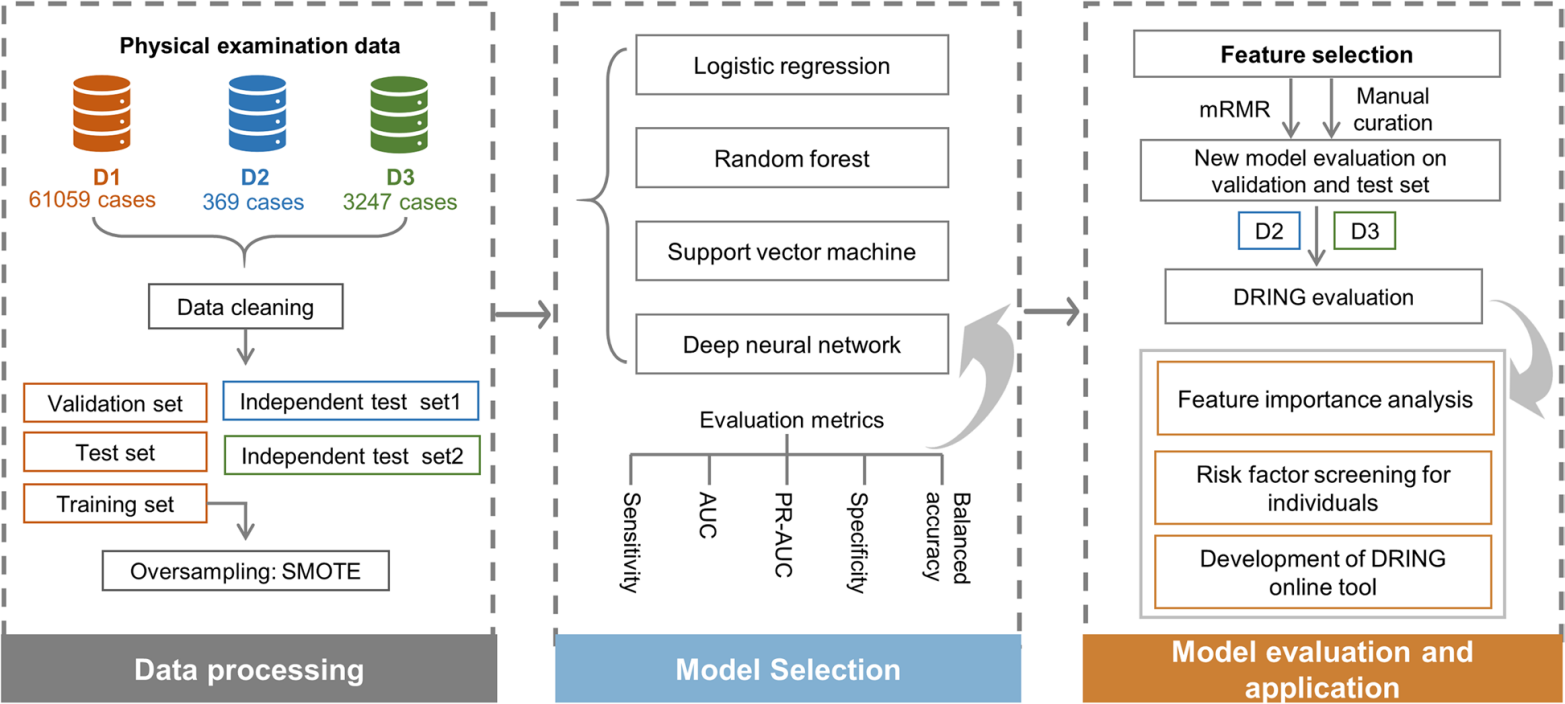
Overview of the DRING approach

With the physical examination data, we presented a computational framework for identifying the diabetic patients with NFG, as shown in Fig. 1. At first, we preprocessed three datasets of D1, D2, and D3 as introduced above, in which D1 was divided into training set, validation set, and test set by 6:1:3, while D2 and D3 as independent test set were used for the evaluation of final model. In view of the class-imbalance of datasets, we used an oversampling method on the training set. Then, multiple widely used machine learning methods including logistic regression (LR), random forest (RF), supported vector machine (SVM), and deep neural network (DNN) were exploited to construct the predictor. Next, we applied feature selection methods on the most superior one of four predictors to improve the feasibility of tool and assessed the performance with independent test sets. Finally, feature importance analysis was used to screen relevant variables with the incidence of diabetes. And we devised a framework for identifying the risk factors of diabetes at individual level and developed an online tool for boosting its clinical practice.

### Model construction

In preliminary, in order to build the predictive model, four machine learning methods were employed, including LR, RF, SVM, and DNN. LR is a variation of linear regression prominently used in classification tasks [23], which finds the best fit to describe the linear relationship between responsible variables and input features and then covert the output to the probability by a sigmoid function. RF is composed of numerous decision trees, which are practically a collection of if–then condition [24] The decision tree recursively split the data into subset based on the best feature and criterion until the stopping criterion is met. In RF, each decision tree is independently trained on random subset of samples and features, which reduces the risk of overfitting. The final decision is voted by all trees improving the overall accuracy and the robustness of the model. SVM, one of the most popular machine learning methods, classifies the samples by finding a hyperplane on the feature space to maximize the margin of points from different classes [25]. It can handle non-linearly separable data by using various kernels such as linear, polynomial, and radial basis function realizing the original feature space into high-dimensional space. The LR, RF, and SVM models were constructed by scikit-learn package in Python 3.8. And default parameters were used in the process of training models. DNN [26] contains input layer, hidden layer, and output layer, where there are plenty of neurons in each layer and the neurons from different layers are connected. For DNN, the connection is generally linear transformation followed by an activation function. Here, we used the ReLU function to activate the linear neurons and softmax function to output the prediction result. In addition, we used the dropout and L2 regularization strategy in the hidden layers to prevent the presence of overfitting. Moreover, the residual blocks also were added into the DNN for simplifying the training process. The DNN was implemented by Pytorch package. In this study, DNN model achieved the best performance when the number of layers at 6 and initial learning rate with 0.0018. Loss on the training set and validation set was depicted in Additional file 1: Fig. S1. And we chose the model with the best performance on validation set for further optimization.

### Model evaluation

Currently, machine learning models for classification task are evaluated by multiple well-established metrics, for example, sensitivity, accuracy, and area under the receiver operating characteristic curve (AUC), etc. Given the seriously unbalanced classes of validation set and test set, here, we exploited sensitivity, specificity, balanced accuracy, AUC, and area under the precision-recall curve (PR-AUC) to evaluate models, which were calculated as following formulas.

1 $$\mathrm{Sensitivity}=\mathrm{TPR}= \frac{TP}{TP+FN}$$

2 $$\mathrm{Specificity}=\mathrm{TNR}=\frac{TN}{TN+FP}$$

3 $$\mathrm{Balanced accuracy}= \frac{TPR+TNR}{2}$$

$TP$, that is, true positive, is the number of correctly classified diabetes patients. $FP$, false positive, denotes the number of normal subjects who were predicted as diabetes. $TN$, true negative, represents the number of correctly classified health subjects. $FN$, false negative, is the number of diabetes patients who were classified as health individuals. And all above metrics range from 0 to 1.

### Feature selection and feature importance analysis

Although the predictive model based on 27 features had a considerable performance, there still exist several possible redundant information or noise features affecting the decision making. To maximize the effective information of features and simplify the model, we used manual curation and max relevance and min redundancy (mRMR) [27] to extract key features for the final model. Towards manual curation, we firstly selected the features with significant difference between the positive samples and the negative samples. To enhance the stability of the predictive model, we removed the features resulting in severe collinearity. As a result, 13 features were retained. For consistency’s sake, the number of feature subset was set to 13 when performing mRMR analysis. In addition, feature selection was executed on the training set for reducing the risk of overfitting. Analysis of feature importance can interpret the prediction model and discover the most relevant features with diabetes. Here, the importance of each feature was measured by its corresponding weight coefficient of the LR model.

### Construction of the DRING webserver

We developed an online tool, DRING (http://www.cuilab.cn/dring), based on the predictive models with 13 features filtered by manual curation and mRMR, where the former is the preferred option. The backend development of website was implemented by Python 2.7, and the interactive pages were constructed on the combination of HTML5, Boostrap 4, and JavaScript.

### Screening the personalized risk factor

Feature importance analysis can help to explain the model; however, it fails to explore the risk factors for incident diabetes at individual level. To find out the potential risk factor for a specific individual, we learnt from the permutation feature importance (PFI) algorithm [24, 28], which is designed for quantifying the importance for each of the variables of a dataset. Here, we adapted PFI to assess the contributions of the features derived from an individual. Specifically, it contains the following 4 steps: (1) given a feature vector, we firstly create a series of random permutation for one of features based on the input dataset; (2) then, we calculate the prediction results for each of new feature vectors; (3) the contribution of the permutated feature is defined as formula 4:

4 $${P =| P}_{r}-\frac{1}{k}{\sum }_{i=1}^{k}{P}_{i} |$$

${P}_{r}$ is the risk score for diabetes calculated with the initial feature vector, here referred to the predictive probability of diabetes; ${P}_{i}$ is the prediction result of i_th_ permutation, and $k$ is the number of permutations; (4) perform the above steps iteratively for each of features. Here, we set *k* to 100,000. The feature with a higher value implies more contribution to the risk of diabetes.

## Results

**Fig. 2:**
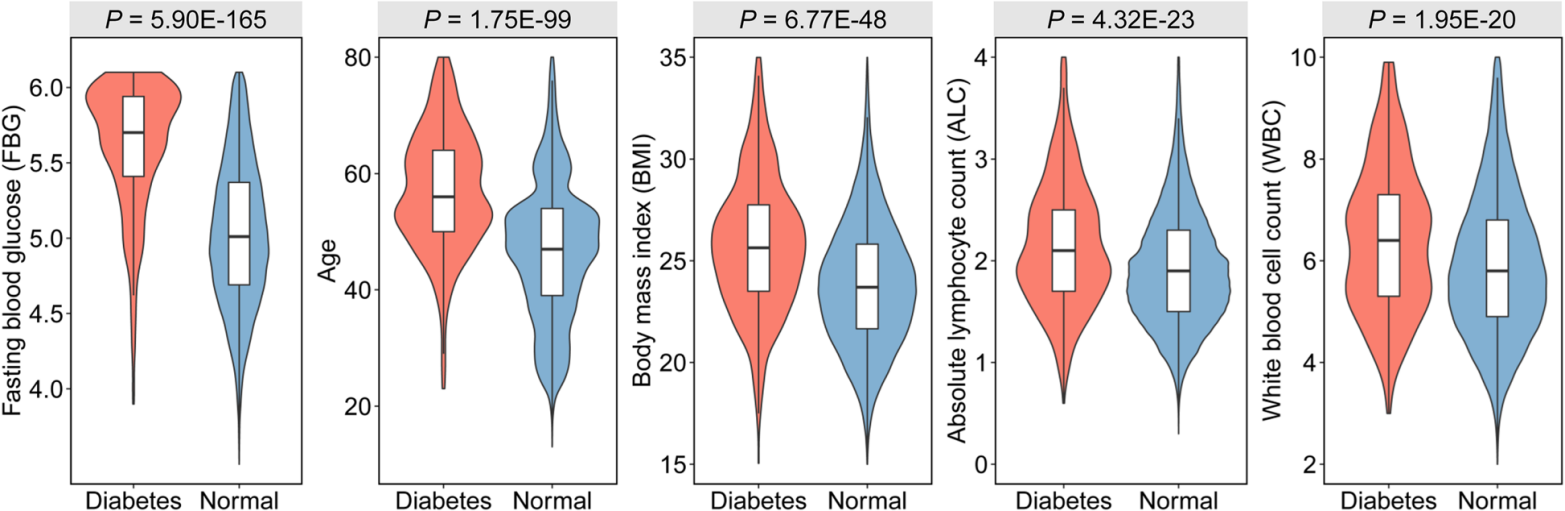
The top 5 features exhibiting the most significant differences between diabetic and non-diabetic individuals with normal fasting glucose

We first collected the physical examination data from First Affiliated Hospital of Wannan Medical College between 2015 and 2018 year, where 61,059 samples with NFG satisfied our inclusion criterion. Nearly 1% (603) of participants were recognized as the diabetes based on the HbA1c level with threshold of 6.5%, and Table 1 depicted the characteristics of NFG individuals with and without diabetes. The diabetes group showed an average BMI higher by 1.08 and an average age older more than 10.6 years compared to the healthy group. The top 5 features with significant difference between diabetes and normal samples were fasting blood glucose (FBG), age, BMI, absolute lymphocyte count (ALC), and white blood cell count (WBC), respectively (Fig. 2), and other 11 features showing significant difference were described in Additional file 1: Fig. S2. The result indicates the considerable potential of demographic and blood test indexes to distinguish diabetes patients in the population with NFG.

**Table 1:** Statistics of characteristics of the diabetic and non-diabetic individuals with and normal fasting glucose

Characteristics	Diabetes	Non-diabetes
*N*	603	60,456
Sex (men %)	58.21%	51.86%
Age	57.49 ± 10.72	46.84 ± 11.93
Height	162.28 ± 8.62	163.77 ± 8.28
Body mass index (BMI)	25.94 ± 3.61	23.86 ± 3.14
Fasting blood glucose (FBG)	5.61 ± 0.43	5.03 ± 0.48
White blood cell count (WBC)	6.72 ± 2.03	6.02 ± 1.7
Neutrophil (NEU %)	57.31 ± 8.9	58.59 ± 8.12
Absolute neutrophil count (ANC)	3.89 ± 1.48	3.57 ± 1.34
Lymphocyte (LYM %)	33.63 ± 8.51	32.69 ± 7.55
Absolute lymphocyte count (ALC)	2.24 ± 1.09	1.93 ± 0.59
Monocyte (MONO %)	6.25 ± 1.64	6.15 ± 1.61
Absolute monocyte count (AMC)	0.42 ± 0.15	0.37 ± 0.13
Eosinophil (EOS %)	2.47 ± 2.13	2.24 ± 1.91
Absolute eosinophil count (AEC)	0.17 ± 0.16	0.14 ± 0.14
Basophil (BASO %)	0.33 ± 0.29	0.33 ± 0.29
Absolute basophil count (ABC)	0.01 ± 0.03	0.01 ± 0.03
Hemoglobin (HGB)	139.5 ± 14.28	140.13 ± 16.29
Hematocrit (HCT)	0.42 ± 0.04	0.42 ± 0.04
Mean corpuscular volume (MCV)	90.05 ± 5.2	90.74 ± 5.23
Mean corpuscular hemoglobin (MCH)	29.81 ± 2.06	30.14 ± 2.11
Red cell distribution width (RDWSD)	13.41 ± 0.91	13.25 ± 1.02
Platelets (PLT)	193.64 ± 62.72	191.83 ± 56.69
Mean platelet volume (MPV)	11.51 ± 1.72	11.37 ± 1.65
Platelet distribution width (PDW)	16.22 ± 1.93	16.14 ± 1.97
Thrombocytopenia (PCT)	0.22 ± 0.06	0.21 ± 0.06
Red blood cell count (RBC)	4.69 ± 0.5	4.66 ± 0.5
Mean corpuscular hemoglobin concentration (MCHC)	330.91 ± 10.04	331.95 ± 11.23

### Comparison of the performance of multiple machine learning methods on prediction of diabetes risk for individuals with NFG

**Fig. 3:**
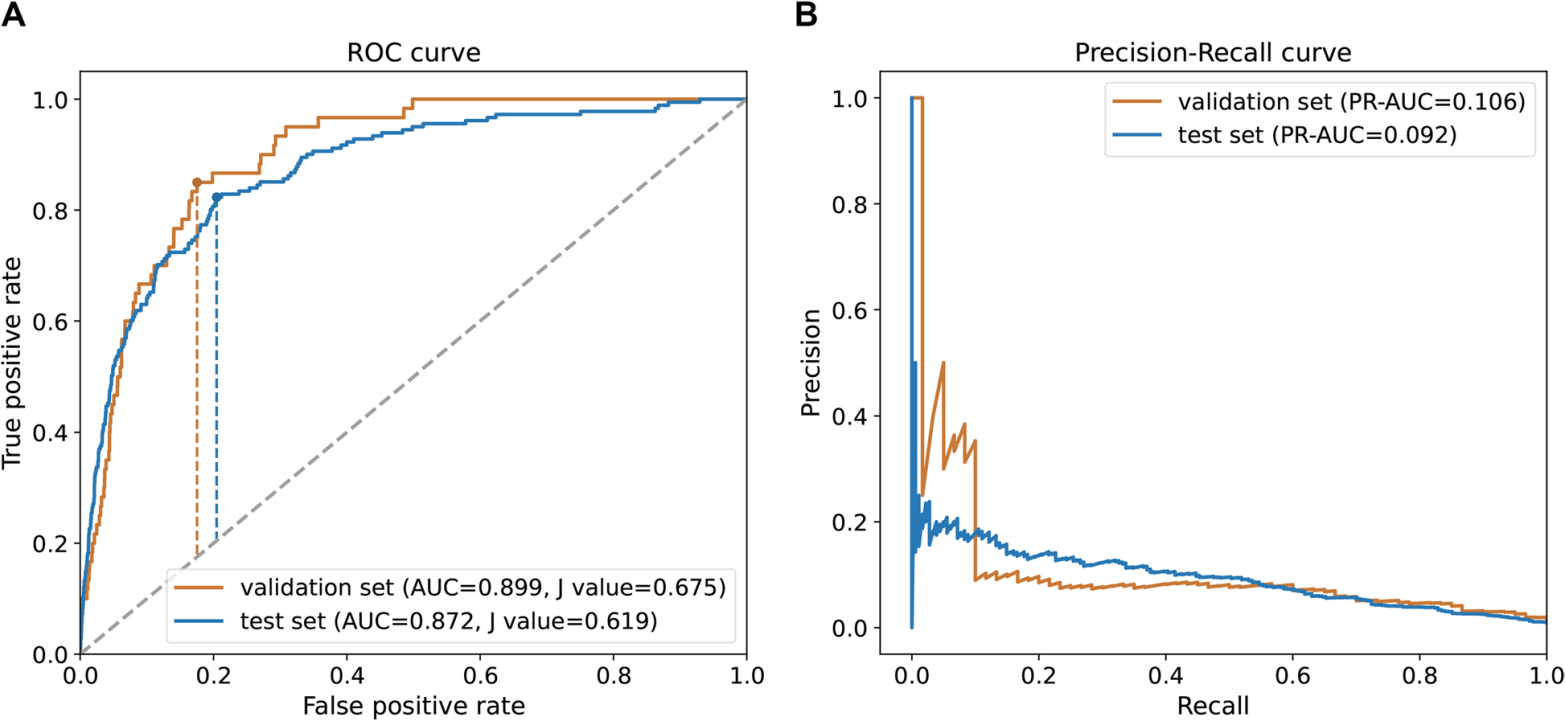
Performance of predictive model on the validation set and the test set from D1 dataset. **A** ROC curve. **B** Precision-recall curve

The performance of multiple machine learning methods including LR, RF, SVM, and DNN on validation set was shown in Table 2. LR model showed the maximum AUC of 0.899 and the PR-AUC of 0.106. We observed a relatively low PR-AUC in all models, which ascribes to the unbalanced class of validation set with over 100-fold discrepancy between diabetes and normal samples. And the PR-AUC of LR was far superior to random performance which equals to the proportion of positive samples. Meanwhile, LR model also significantly outperformed the others in respect of sensitivity at 85.0% and balanced accuracy scores at 83.0% while RF displayed the highest specificity score (99.9%). To maximize the identification of diabetes patients, the LR algorithm were finally selected for further constructing the predictive model. The results of fivefold cross-validation on the training set showed an AUC of 0.906, PR-AUC of 0.879, sensitivity of 85.9%, and balanced accuracy of 83.4%. Furthermore, the LR model also showed a reliable performance (AUC = 0.872, PR-AUC = 0.092) on the test set, as shown in Fig. 3A and B. The sensitivity and balanced accuracy on test set was 77.9% and 79.5%, respectively. The results suggested the potential of demographics and blood routine indexes on discriminating the diabetic patients and normal individuals with NFG.

**Table 2:** Comparison of performance of four machine learning methods

	**AUC**	**PR-AUC**	**Recall**	**Specificity**	**Balanced accuracy**
RF	0.860	0.071	0.017	0.999	0.508
SVM	0.801	0.051	0.333	0.942	0.638
DNN	0.844	0.082	0.417	0.948	0.685
LR	0.899	0.106	0.850	0.811	0.830

The values of AUC, PR-AUC, recall, specificity, and balanced accuracy are range from 0 to 1, with a higher value indicating a better performance

*RF* random forest, *SVM* supported vector machine, *DNN* deep neural network, *LR* logistic regression, *AUC* area under the curve of receiver operating characteristic, *PR-AUC* are under the curve of precision-recall

### Feature selection for constructing the final classifier

**Fig. 4:**
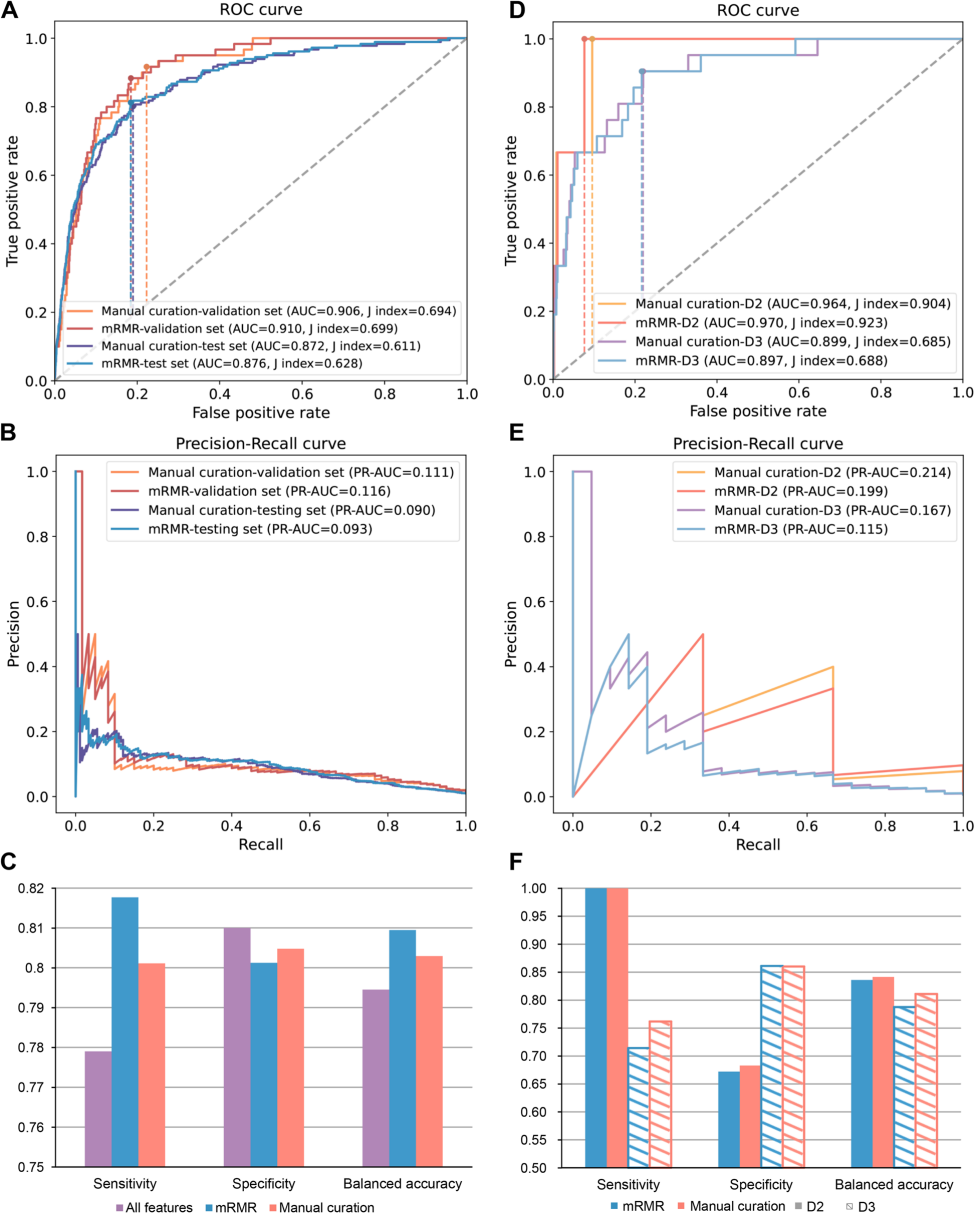
Feature selection for the final model using mRMR and manual curation. **A** ROC curve of the models constructed by mRMR- and manual curation- selected features on the validation set and the test set of dataset D1. **B** Precision-recall curve of above models. **C** Comparison of sensitivity, specificity, and balanced accuracy on test set between the model constructed before and after feature selection. **D** ROC curve of models using selected features on the two newly recruited independent test sets. **E** Precision-recall curve of above models. **F** Comparison of other metrics on the two newly recruited independent test sets

A high correlation of several pairs of features has been observed (Additional file 1: Fig. S3), for example, hemoglobin (HGB) and hematocrit (HCT), neutrophil (NEU), and lymphocyte (LYM), which could introduce the redundant information affecting the decision making and stability of the model. Thus, manual curation and max relevance and min redundancy (mRMR) were applied to search for an optimal feature space. As a result, half of the features (i.e., 13 features) from manual curation and mRMR were hold to re-construct the LR models, respectively (Table 3), where 9 shared features were observed (*P*-value = 0.041, Fisher’s exact test). The top 4 features selected by manual curation and mRMR are consistent, which are FBG, BMI, absolute lymphocyte count (ALC), and age. For either method, the model constructed with 13 features showed slight improvement compared with that of with 27 features on validation set, achieving the AUC of 0.906 and PR-AUC of 0.111 on manual curation and the AUC of 0.910 and PR-AUC of 0.116 on mRMR (Fig. 4A and B). And on test set, the AUC and PR-AUC of the model were 0.872 and 0.090 by manual curation and 0.876 and 0.093 by mRMR with moderately advancement than that of 27 features. Similarly, the models established with two methods both increased the sensitivity and balanced accuracy on test set, with maximum 3.9% increasement and maximum 1.4% increasement, respectively (Fig. 4C). Then, we further evaluated the models with two newly recruited independent test sets (D2 and D3). As a result, the AUC values of two models were both over 0.95 on D2 and were nearly 0.90 on D3 (Fig. 4D). Moreover, we found a high Youden’s index, also called as J index, on D2 with 0.904 by manual curation and 0.923 by mRMR. The model based on manual curation, in which PR-AUC is 0.214 on D2 and 0.167 on D3, performed better than that of mRMR, with the PR-AUC of 0.199 on D2 and 0.115 on D3 (Fig. 4E). As to sensitivity, specificity, and balanced accuracy, the model based on manual curation also overwhelmed that based on mRMR (Fig. 4F). We observed that the worst false positive rate was 31.7% performed by the model of mRMR on D2, which only comprised 369 samples with three diabetes patients (the positive sample) and represented an extremely imbalanced condition. Other specificity scores were all over 80.0%. Together, the model displayed superior performance on the independent test sets, which demonstrated the core features are sufficiently outstanding to detect the missed diabetic patients in NFG population.

**Table 3:** The subset of features for the final model selected by mRMR and manual curation

mRMR	Manual curation	Rank
Fasting blood glucose (FBG)	Fasting blood glucose (FBG)	1
Absolute lymphocyte count (ALC)	Age	2
Age	Body mass index (BMI)	3
Body mass index (BMI)	Absolute lymphocyte count (ALC)	4
Eosinophil (EOS)	White blood cell count (WBC)	5
Red cell distribution width (RDW)	Absolute monocyte count (AMC)	6
Platelets (PLT)	Red cell distribution width (RDW)	7
White blood cell count (WBC)	Absolute eosinophil count (AEC)	8
Height	Mean corpuscular hemoglobin (MCH)	9
Neutrophil (NEU)	Sex	10
Basophil (BASO)	Height	11
Mean corpuscular hemoglobin concentration (MCHC)	Neutrophil (NEU)	12
Mean corpuscular volume (MCV)	Mean corpuscular volume (MCV)	13

### Feature importance ranking

**Fig. 5:**
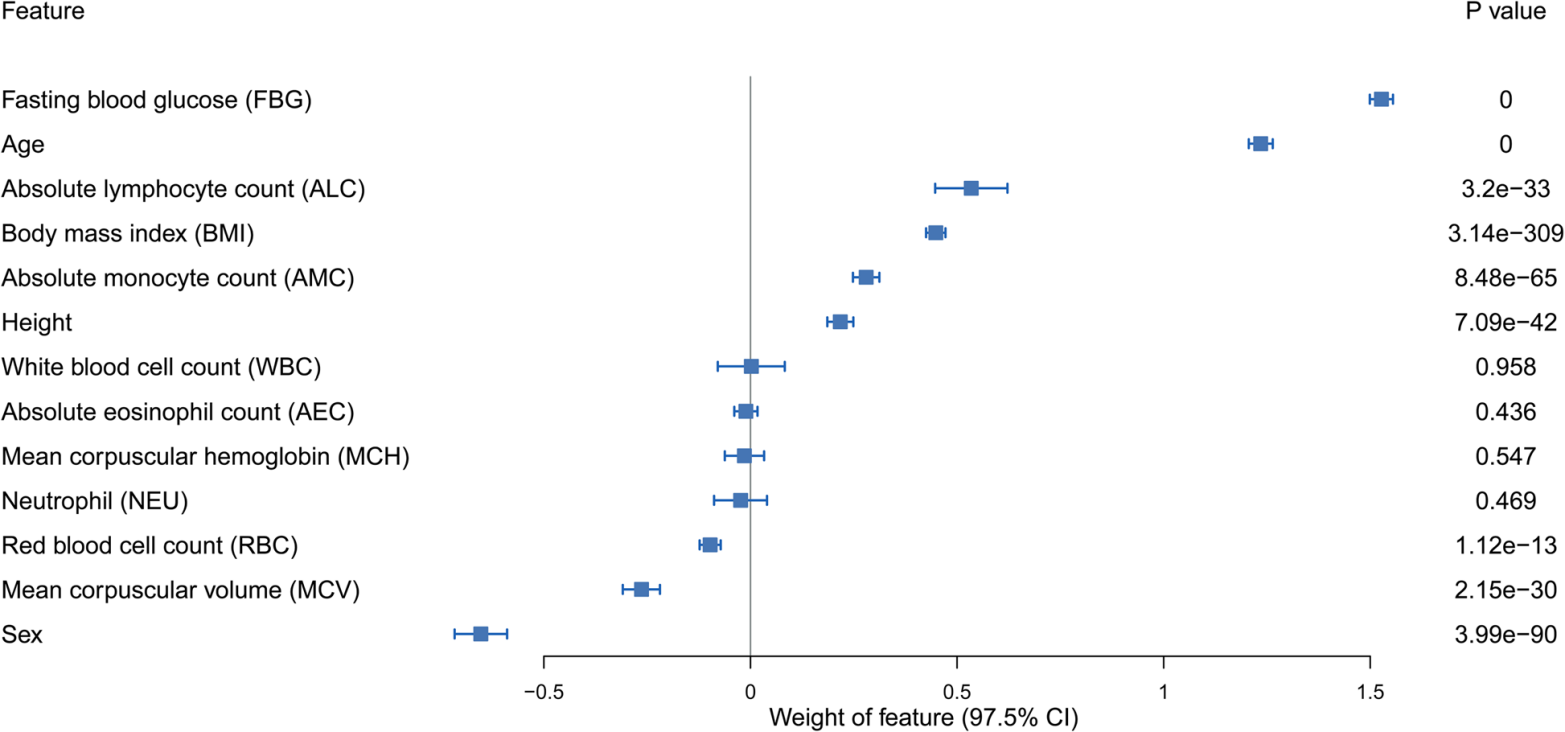
Feature importance ranking of the models constructed by the features from manual curation method

To explore which features contribute most to the diabetic risks, we used the weights in LR model constructed with 13 features from manual curation to rank the features, as described in Fig. 5. And the feature importance of the predictor based on mRMR was depicted in Additional file 1: Fig. S4. As a result, the top 5 variables were FBG, age, sex, ALC, and BMI. Previous studies have demonstrated that diabetes risk increases as FBG level increases even within the normal range [29], thereby the process of decision making depends heavily on the FBG level although all data were only derived from the samples with NFG. Moreover, it is well established that age and BMI are risk factors for the diabetes occurrence. Interestingly, we observed that sex had a high importance value even more than BMI, which suggests an apparent difference on the risk of diabetes between men and women. In addition, ALC, absolute monocyte count (AMC), and mean corpuscular volume (MCV) also showed the moderately predictive capability of the risk for diabetes in population with NFG. Neutrophil (NEU) and white blood cell count (WBC) also showed relative higher weights on the model with mRMR.

### Screening of personalized risk factors

**Fig. 6:**
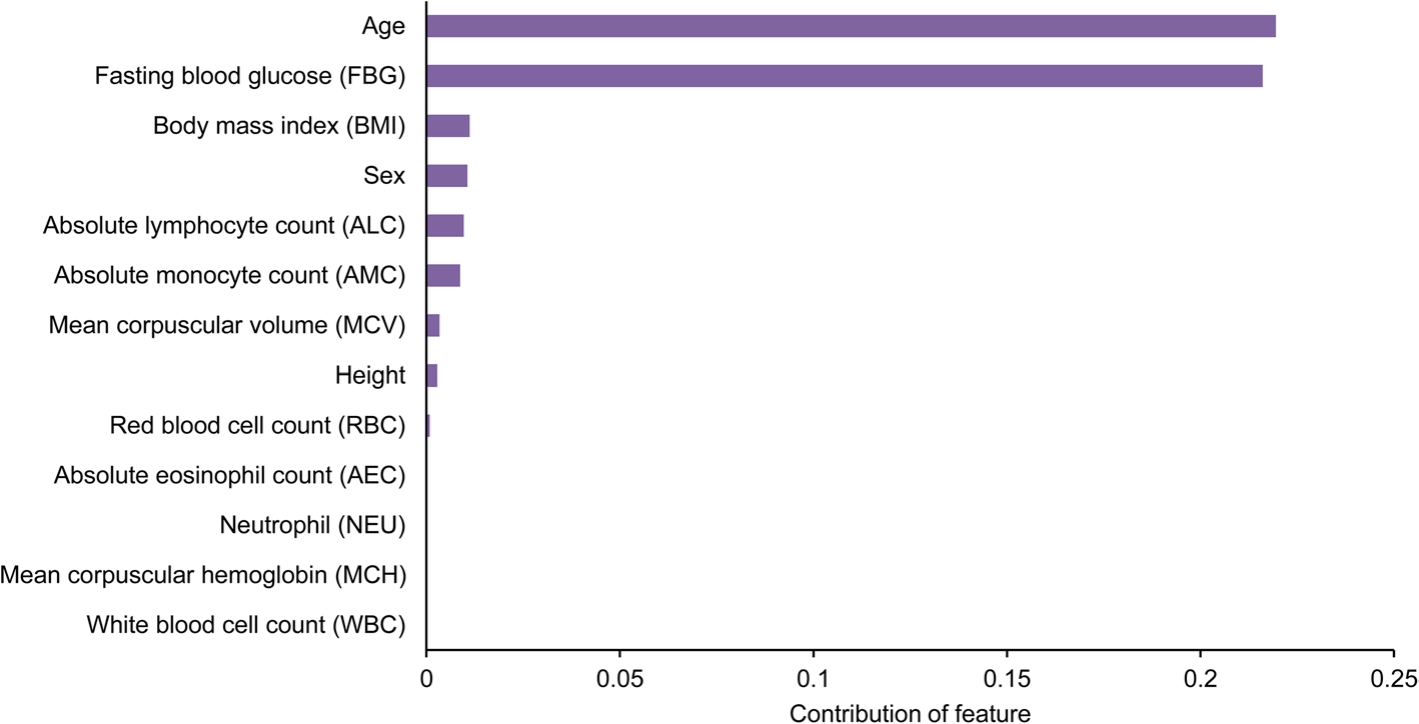
Screening of risk factors for incident diabetes on a case study

Then, we developed a framework to reveal the diabetic risk factors at individual level based on the principle of permutation feature importance (PFI), which would guide the personalized early intervention. The selected case was from the external validation set-2, who showed the characteristic as following, absolute eosinophil count (AEC): 0.23, age: 69, ALC: 2.29, AMC: 0.51, BMI: 26.04, FBG: 5.76, height: 158.0, mean corpuscular hemoglobin (MCH): 29.7, MCV: 89, NEU: 56.50, red cell distribution width (RDW): 13, sex: female, and WBC: 7.05. And this individual was predicted as a diabetic patient correctly, with the probability of 0.977. To explore her risk factor of diabetes, we calculated the contribution of all features as described in Fig. 6. As a result, the major risk factors for her incident diabetes are age, FBG, and BMI, although her FBG level was seemingly pretty normal. In addition, we noted that higher age and BMI indeed increasing her diabetes risk. The result provides the indexes need to be preferentially intervened for individuals. Finally, we have ensembled this module in the DRING webserver for ease of application.

## Discussion

Considering that there exists a small proportion of diabetes patients with normal fasting glucose level who could be missed during screening, we assessed the ability of common risk factors to detect the missed diabetic patients in population with normal fasting glucose (NFG) and thus constructed DRING, a machine learning model for predicting the diabetic individuals with NFG based on physical examination data of over 60,000 samples derived from three Chinese cohorts. DRING showed more than 0.9 of AUC and 75% of sensitivity on newly recruited independent test set. For enhancing the interpretability of DRING, potential characteristics related to diabetes except for fasting blood glucose contains BMI, age, ALC, and sex by feature importance ranking analysis. Moreover, the analysis for exploring the personalized risk factors of diabetes was proved to be practical with a case study. Two models and the framework of screening personalized risk factors were integrated into DRING webserver, which requires only 13 features as input to predict the risk of diabetes. To accommodate different requirements for specificity levels, the webserver offers users a range of choices to customize the specificity levels.

With the increase of data volume, machine learning techniques have the potential to revolutionize diabetes screening by enabling more accurate risk stratification and timely interventions. The performance of ML-based methods is influenced by the availability of the number of samples and features. For instance, the combination of ML techniques and electronic health record data could enhance the effectiveness of diabetes screening and improving patient outcomes. One of the challenging issues in utilizing ML techniques is selecting the most suitable method to achieve optimal performance on a given dataset. Nearly all machine learning techniques have been applied on the diabetes risk prediction [16], while no single method that consistently outperforms other methods across diverse datasets. Dinh et al. reported that the model of predicting diabetes with eXtreme Gradient Boost (XGBoost) performed best than those of RF, SVM, and LR based on the National Health and Nutrition Examination Survey (NHANES) dataset [30]. With the Pima Indian Diabetes Database (PIDD), which is a widely used dataset in diabetes recognition with machine learning, a study constructed 24 classifiers such as decision tree, LR, discriminant analysis, k-nearest neighbors, and ensemble learners and found the best accuracy score of 77.9% was produced by the LR model [31]. Jahangir and his colleagues devised automatic multilayer perceptron model achieving an accuracy of 88.7% on PIDD [32], while they did not train other machine learning models using processed data. The difference in performance between these two studies may not solely depend on the ability of machine learning models but also the dataset partitioning and preprocessing could impact the results. A recent study conducted a systematic analysis among 71 studies of clinical prediction models and concluded that no evidence of superior performance of other machine learning methods over LR [33]. Our results have also shown that LR model obtains a higher AUC and balanced accuracy in identifying the diabetes from population with NFG. This could be attributed to the fact that complex models are not suitable when using a limited number of features. The factors, such as the size of the dataset, interpretability, and the balance of precision and complexity, collectively determine the optimal choice of models.

There is typically no specific guideline that needs to be strictly followed when employing artificial intelligence models for diabetes screening, except for the initial step of sample definition, that is, defining positive and negative samples. The definition of normal fasting glucose level varies among different guidelines, for example, the American Diabetes Association (ADA) uses FBG of 5.6 mmol/L as the definition of normal fasting glucose, while the threshold is set to 6.1 mmol/L according to the WHO or International Diabetes Federation (IDF). Here, we screened the samples with NFG by the criterion of WHO and found the fasting blood glucose (FBG) level for most diabetes patients is approximate to 6.0 (Fig. 1A). In our cohorts, it was observed that the number of diabetes patients sharply decreased when the threshold for NFG was lowered. Specifically, less than half of the individuals were classified as diabetes when NFG level was set at 5.69 (Additional file 1: Fig. S5). Accordingly, we recommended the FBG of 5.69 as the alarming line of diabetes for Chinese, that is, a person should notice the sugar intake and go for a thorough diabetes-focused examination in case his fasting glucose is more than 5.69. Although it was questioned when the justification for lowering the threshold of normal fasting glucose recommended by ADA [34], the warning line of normal fasting glucose needs timely adjustment for people from different races or regions considering the significant difference in diet, lifestyle, and environment.

The risk factors are diverse for incident diabetes. We employed clinical basic information and blood routine test indicators to train the prediction model. In contrast, most other methods for prediction the risk of diabetes rely on the basic information including diabetes family history and blood pressure combined with biochemical markers such as triglycerides and total cholesterol [15, 35, 36] and rarely include the features of blood routine test. Except for the well-known factors such as BMI and age [35, 37], the analysis of feature importance ranking showed that absolute lymphocyte count (ALC), mean corpuscular volume (MCV), white blood cell count (WBC), and neutrophil (NEU) also are important for identifying the diabetes patients (Fig. 5 and Additional file 1: Fig. S4). Twig et al. had revealed that WBC was an independent risk factor for incident diabetes in young men [38]. The inclusion of more diabetes-associated variables and higher resolution data is likely to improve the accuracy of the predictive model. Quincy et al. presented a diabetes risk stratification model integrating physiological, biochemical, and genomics data and achieved superior testing accuracies [39]; however, its practicality is weakened due to the difficulty in data acquisition.

The strength of this study lies in that it is the first prediction model specially designed to identify diabetes patients who are at high risk of being missed according to our knowledge, which serves as a valuable supplement to existing diabetes risk prediction models. The model has also been integrated into the online tool, facilitating its potential clinical application. However, a key limitation of this study is that it is challenging to assert the generalization of the prediction model on global population. Diabetes is influenced by various factors such as race and environment, although our method was validated and tested on multiple cohorts, all of which were Chinese populations; thus, its performance in other populations remains uncertain. The generalization of the current method needs to be assessed through more external validation datasets, especially those involving other ethnic populations. Secondly, there exist various prediction models for diabetic risk assessment at present, but it is still incomparable between DRING and other methods because DRING is extensively used for distinguishing the diabetes patients with NFG and healthy individuals. Third, the precision of current method is relatively low, which is markedly impacted by the severely imbalanced distribution of diabetes and normal individuals. The ratio of positive to negative samples is over 1:100. Thus, even with the inclusion of over 60,000 samples in our study, the number of diabetes samples is only around 600. It is undoubtable that this scenario aligns with the real-world condition represented by infrequent cases of diabetes patients with NFG and major healthy individuals with NFG. In the future, the model of predicting diabetes risk for the population with NFG introducing more crucial features such as waist-to-hip ratio, blood pressure, and common biochemical indicators might enhance its precision. Last but not the least, conducting a comprehensive health technology assessment is necessary to promote our method serving as a decision-making support system in diabetes diagnosis.

## Conclusions

The present study assessed the ability of common physical examination indexes to stratify the risk of diabetes and provided a prediction tool called DRING for identifying the diabetes individuals with normal fasting glucose based on routine clinical information. The outcome obtained from three independent cohorts indicates the clinical reliability of DRING in the future, which allows early diagnosis and interventions for those individuals most likely to be missed and thus improves care and management of diabetes.

### Supplementary Information

**Additional file 1: Fig. S1.** Training and validation loss of DNN. **Fig. S2.** The other 11 characteristics with significant differences between diabetic and non-diabetic individuals with normal fasting glucose. * *P* < 0.05, ** *P* < 0.01, *** *P* < 0.001, **** *P* < 0.0001. **Fig. S3.** Correlation of all features in the training set. **Fig. S4.** Feature importance ranking of the models constructed by mRMR-selected features. **Fig. S5.** Number of diabetic patients towards different thresholds of normal fasting glucose. Orange point is a turning point that the number of individuals with diabetes has halved when using 5.69 as the threshold of normal fasting glucose.

## Data Availability

Data cannot be published without patients’ consent. Researchers who are interested for academic need could contact the corresponding authors.

## Abbreviations

ABC: Absolute basophil count
ADA: American Diabetes Association
AEC: Absolute eosinophil count
ALC: Absolute lymphocyte count
AMC: Absolute monocyte count
ANC: Absolute neutrophil count
BASO: Basophil
BMI: Body mass index
DM: Diabetes mellitus
DNN: Deep neural network
DRING: Diabetes risk of individuals with normal fasting glucose
DRS: Diabetes risk score
EOS: Eosinophil
FBG: Fasting blood glucose
HCT: Hematocrit
HGB: Hemoglobin
IDF: International Diabetes Federation
LR: Logistics regression
LYM: Lymphocyte
MCH: Mean corpuscular hemoglobin
MCHC: Mean corpuscular hemoglobin concentration
MCV: Mean corpuscular volume
MONO: Monocyte
mRMR: Max relevance and min redundancy
MPV: Mean platelet volume
NEU: Neutrophil
NFG: Normal fasting glucose
NHANES: National Health and Nutrition Examination Survey
PCT: Thrombocytopenia
PDW: Platelet distribution width
PFI: Permutation feature importance
PLT: Platelets
RBC: Red blood cell count
RDW: Red cell distribution width
RF: Random forest
SMOTE: Synthetic minority over-sampling technique
SVM: Supported vector machine
WBC: White blood cell count
WHO: World Health Organization
