# Supplementary figures and images for: Detection of diabetic patients in people with normal fasting glucose using machine learning
Source: BMC Med. 2023 Sep 7;21:342. doi: 10.1186/s12916-023-03045-9 (PMC10483877; doi:10.1186/s12916-023-03045-9)

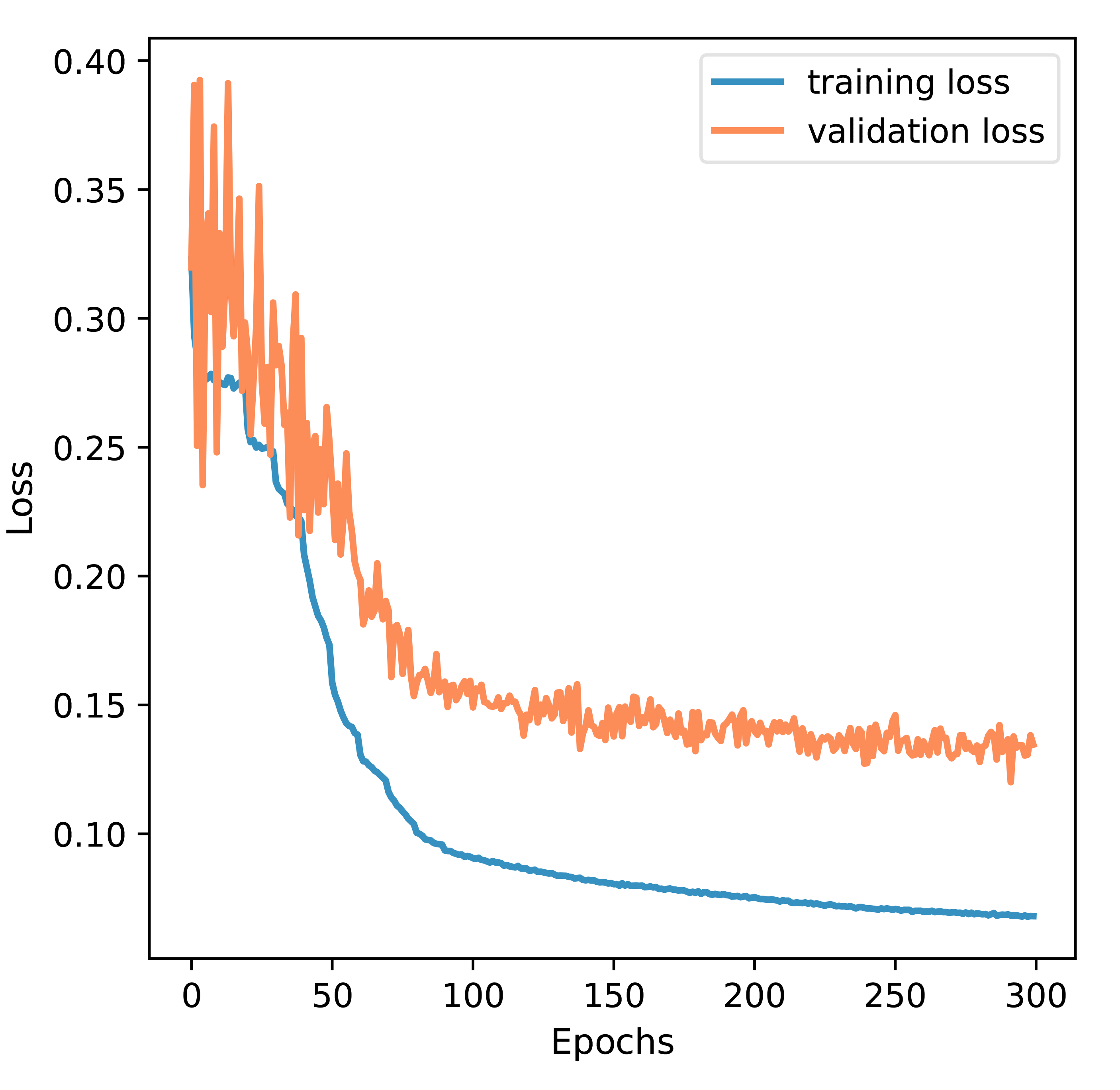

Supplement: Supplementary file 1 — Additional file 1: Fig. S1. Training and validation loss of DNN. Fig. S2. The other 11 characteristics with significant differences between diabetic and non-diabetic individuals with normal fasting glucose. * P < 0.05, ** P < 0.01, *** P < 0.001, **** P < 0.0001. Fig. S3. Correlation of all features in the training set. Fig. S4. Feature importance ranking of the models constructed by mRMR-selected features. Fig. S5. Number of diabetic patients towards different thresholds of normal fasting glucose. Orange point is a turning point that the number of individuals with diabetes has halved when using 5.69 as the threshold of normal fasting glucose. [file 12916_2023_3045_MOESM1_ESM.zip › Figure S1.tif]

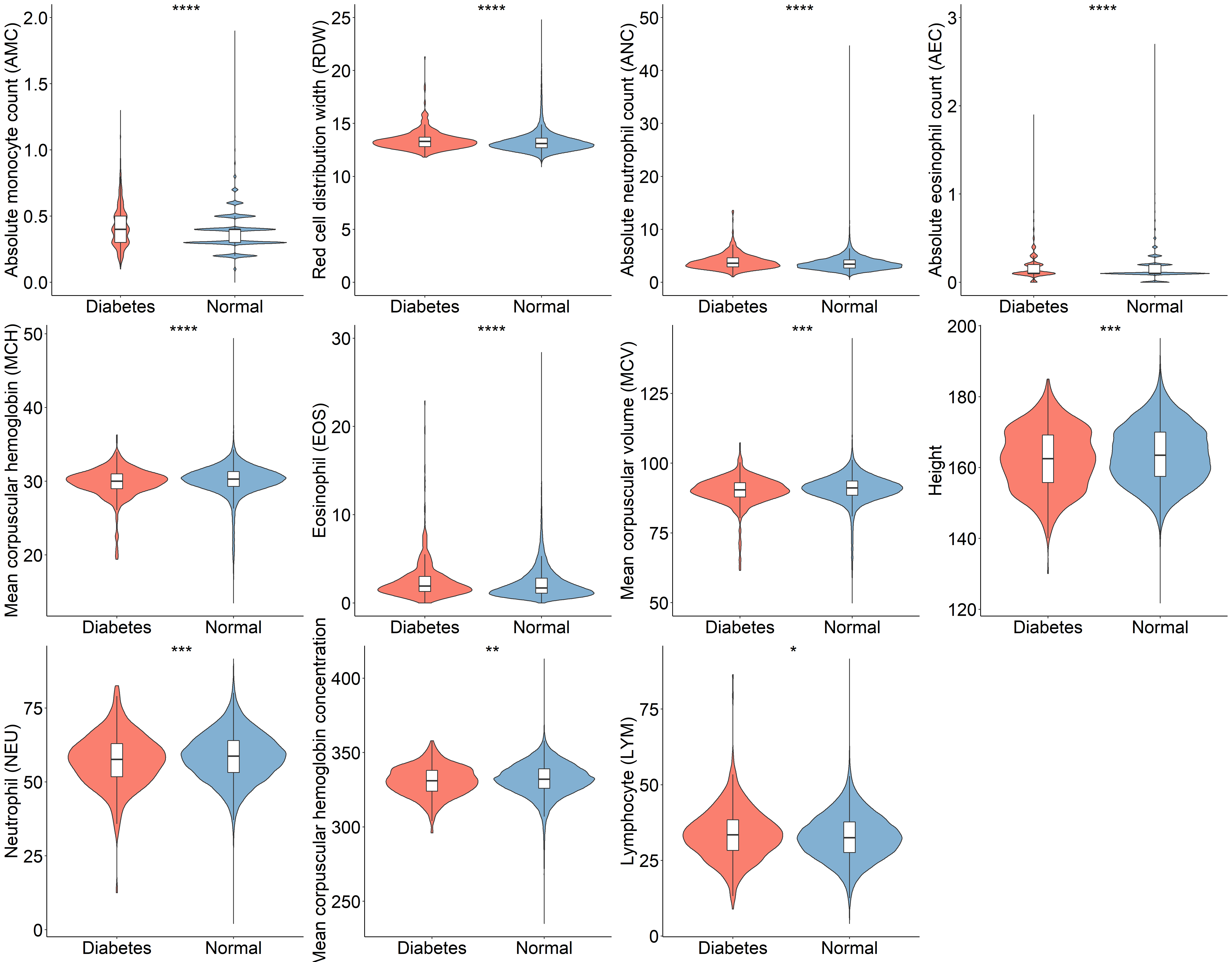

Supplement: Supplementary file 1 — Additional file 1: Fig. S1. Training and validation loss of DNN. Fig. S2. The other 11 characteristics with significant differences between diabetic and non-diabetic individuals with normal fasting glucose. * P < 0.05, ** P < 0.01, *** P < 0.001, **** P < 0.0001. Fig. S3. Correlation of all features in the training set. Fig. S4. Feature importance ranking of the models constructed by mRMR-selected features. Fig. S5. Number of diabetic patients towards different thresholds of normal fasting glucose. Orange point is a turning point that the number of individuals with diabetes has halved when using 5.69 as the threshold of normal fasting glucose. [file 12916_2023_3045_MOESM1_ESM.zip › Figure S2.tif]

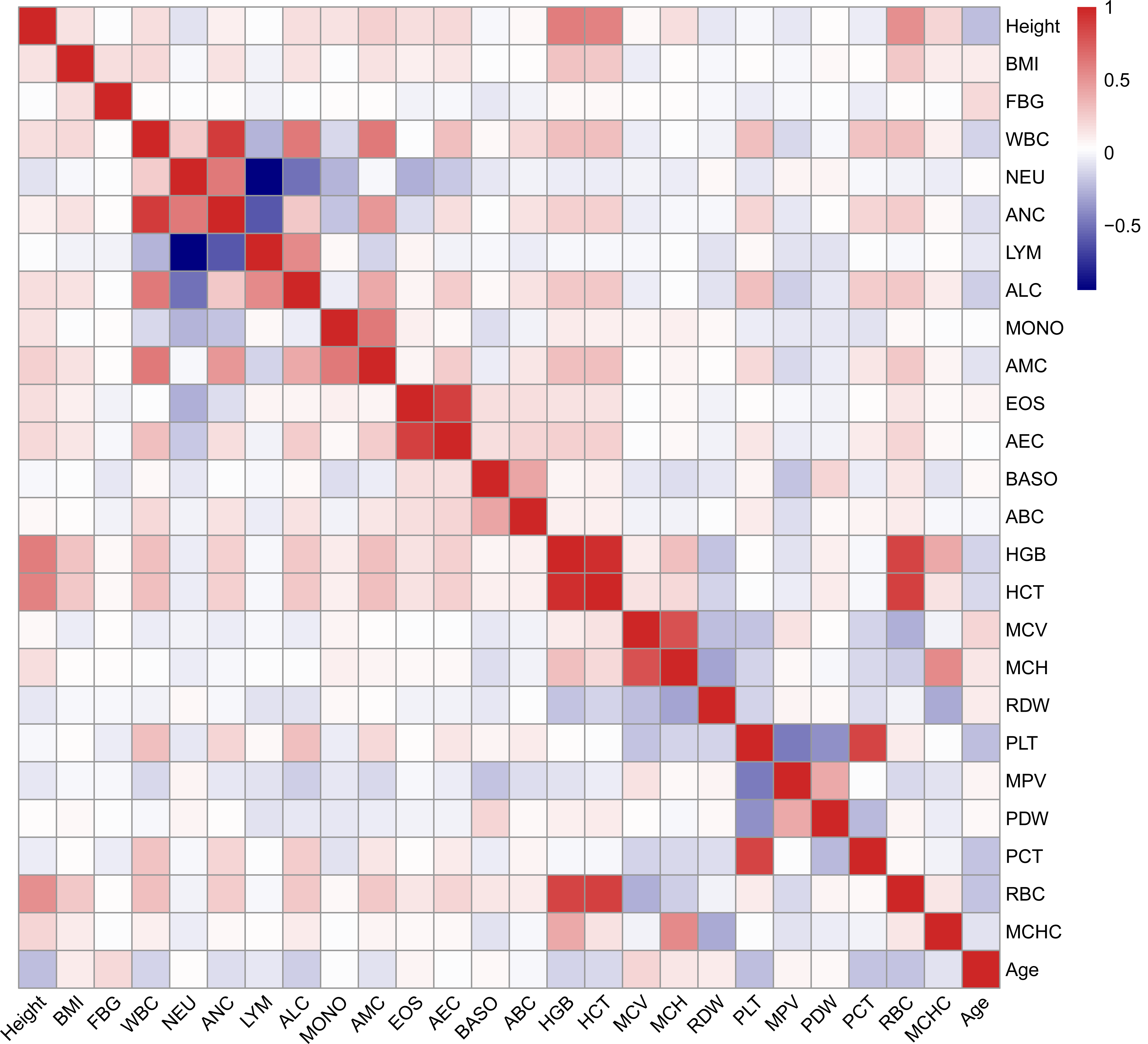

Supplement: Supplementary file 1 — Additional file 1: Fig. S1. Training and validation loss of DNN. Fig. S2. The other 11 characteristics with significant differences between diabetic and non-diabetic individuals with normal fasting glucose. * P < 0.05, ** P < 0.01, *** P < 0.001, **** P < 0.0001. Fig. S3. Correlation of all features in the training set. Fig. S4. Feature importance ranking of the models constructed by mRMR-selected features. Fig. S5. Number of diabetic patients towards different thresholds of normal fasting glucose. Orange point is a turning point that the number of individuals with diabetes has halved when using 5.69 as the threshold of normal fasting glucose. [file 12916_2023_3045_MOESM1_ESM.zip › Figure S3.tif]

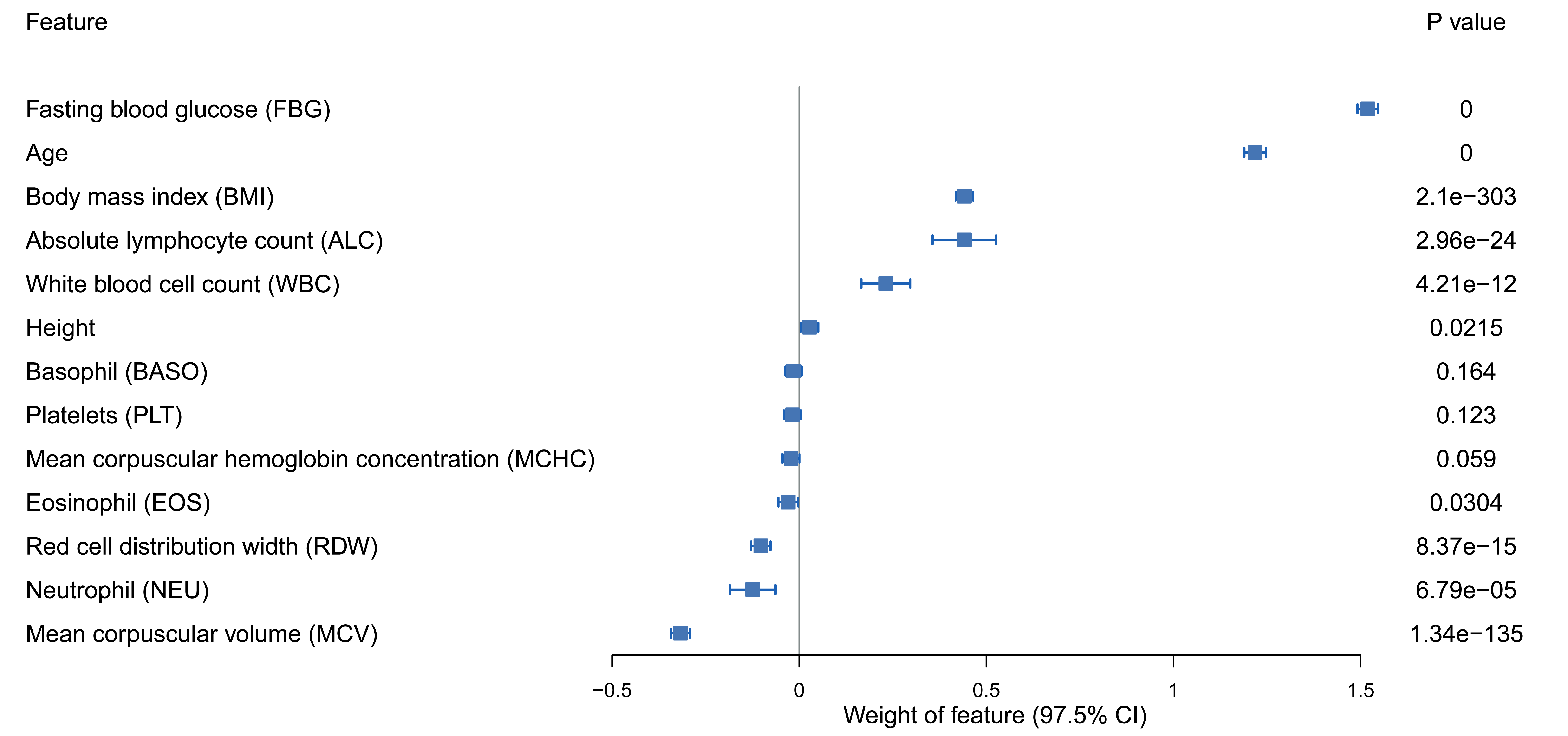

Supplement: Supplementary file 1 — Additional file 1: Fig. S1. Training and validation loss of DNN. Fig. S2. The other 11 characteristics with significant differences between diabetic and non-diabetic individuals with normal fasting glucose. * P < 0.05, ** P < 0.01, *** P < 0.001, **** P < 0.0001. Fig. S3. Correlation of all features in the training set. Fig. S4. Feature importance ranking of the models constructed by mRMR-selected features. Fig. S5. Number of diabetic patients towards different thresholds of normal fasting glucose. Orange point is a turning point that the number of individuals with diabetes has halved when using 5.69 as the threshold of normal fasting glucose. [file 12916_2023_3045_MOESM1_ESM.zip › Figure S4.tif]

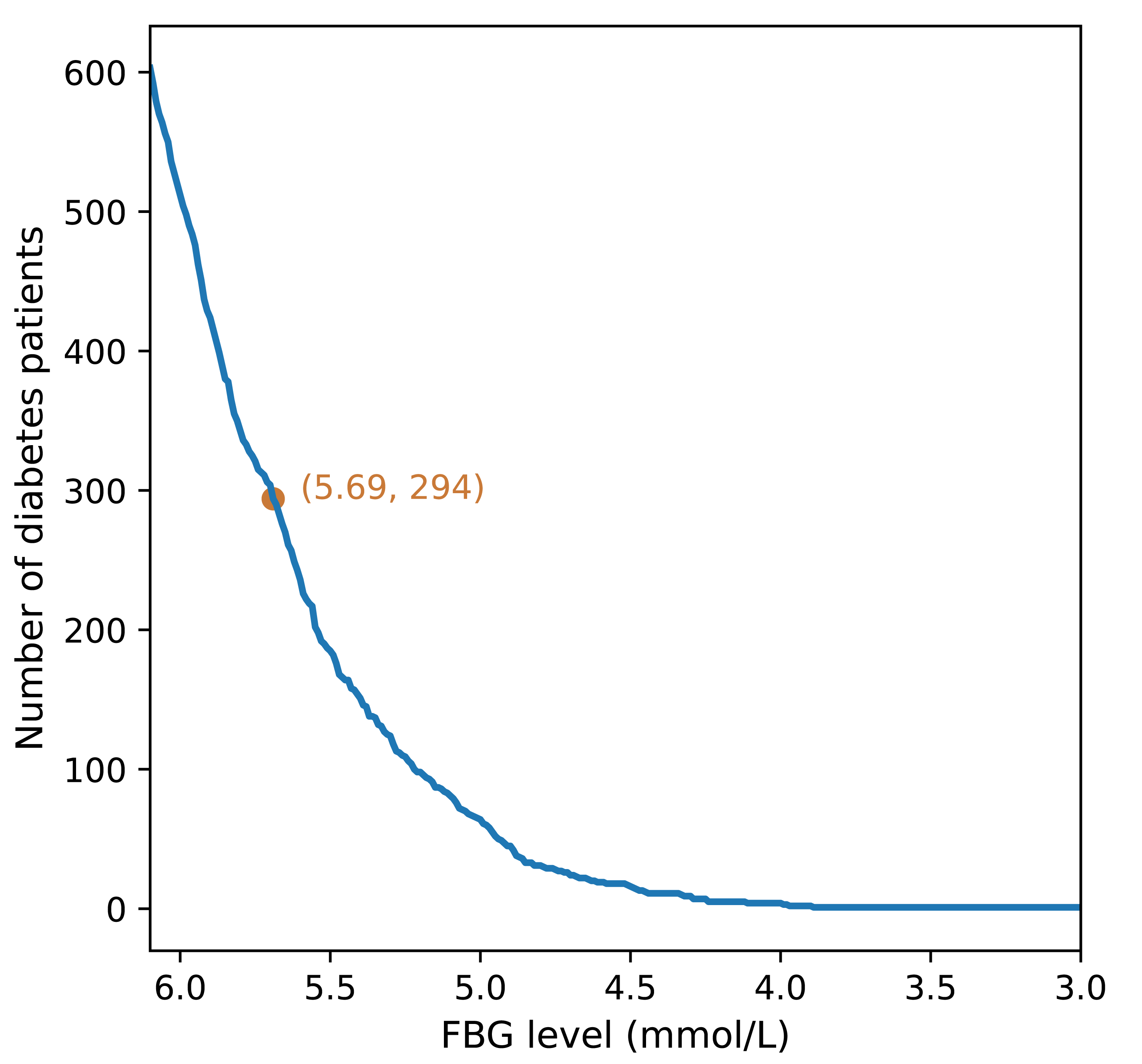

Supplement: Supplementary file 1 — Additional file 1: Fig. S1. Training and validation loss of DNN. Fig. S2. The other 11 characteristics with significant differences between diabetic and non-diabetic individuals with normal fasting glucose. * P < 0.05, ** P < 0.01, *** P < 0.001, **** P < 0.0001. Fig. S3. Correlation of all features in the training set. Fig. S4. Feature importance ranking of the models constructed by mRMR-selected features. Fig. S5. Number of diabetic patients towards different thresholds of normal fasting glucose. Orange point is a turning point that the number of individuals with diabetes has halved when using 5.69 as the threshold of normal fasting glucose. [file 12916_2023_3045_MOESM1_ESM.zip › Figure S5.tif]
